# On Hamiltonian Decomposition Problem of 3-Arc Graphs

**DOI:** 10.1155/2022/5837405

**Published:** 2022-04-28

**Authors:** Guangjun Xu, Qiang Sun, Zuosong Liang

**Affiliations:** ^1^School of Mathematics, Zunyi Normal University, Zunyi, Guizhou, China; ^2^School of Mathematical Science, Yangzhou University, Yangzhou, China; ^3^School of Management, Qufu Normal University, Rizhao, China

## Abstract

A 4-tuple (*y*, *x*, *v*, *w*) in a graph is a 3-arc if each of (*y*, *x*, *v*) and (*x*, *v*, *w*) is a path. The 3-arc graph of *H* is the graph with vertex set all arcs of *H* and edge set containing all edges joining *xy* and *vw* whenever (*y*, *x*, *v*, *w*) is a 3-arc of *H*. A Hamilton cycle is a closed path meeting each vertex of a graph. A graph *H* including a Hamilton cycle is called Hamiltonian and *H* has a Hamiltonian decomposition provided its edge set admits a partition into disjoint Hamilton cycles (possibly with a single perfect matching). The current paper proves that every connected 3-arc graph consists of more than one Hamilton cycle. Since the 3-arc graph of a cubic graph is 4-regular, it further proves that each 3-arc graph of a cubic graph in a certain family has a Hamiltonian decomposition.

## 1. Introduction

A path (cycle) in a graph passing each vertex is said to be a *Hamilton path (cycle)*. A graph is *hamiltonian* if it consists of a Hamilton cycle and is *Hamilton-connected* if every pair of vertices are joined by a Hamilton path. A partition of the edge set *E*(*G*) into Hamilton cycles (or plus a perfect matching when *G* is *k*-regular with *k* odd) is called a *Hamiltonian decomposition* of *G*. The Hamiltonian problems and related topics are classical in combinatorics and have been received extensive treatment. See [1–3] for more results on this area of investigation.

The current paper concentrates on the class of 3-arc graphs. This class of graphs is produced from a graph operation named 3-arc graph operation, which is similar to a line graph operation. This operation was initially introduced in [4] in exploring certain families of imprimitive symmetric graphs. It was also exploited in investigating several classes of symmetric graphs. See [4, 5] and references therein.

We consider only a graph with finite order and undirected without loops. The term *multigraph* is used if there are multiple edges connecting the same pair of vertices. An *arc* of *H*=(*V*(*H*), *E*(*H*)) is a directed edge. If *x*, *y* are adjoined in *H*, we write *xy* to denote the directed edge outgoing from *x* to *y*, *yx*(≠*xy*) the directed edge from *y* to *x*, and {*x*, *y*} the edge connecting *x* and *y*. A 4-tuple (*y*, *x*, *v*, *w*) in a graph is a *3-arc* if each of (*y*, *x*, *v*) and (*x*, *v*, *w*) is isomorphic to a path on three vertices. It may happen that *y*=*w*. Suppose *H* is a multigraph. A *walk* in *H* of length *t* is as follows:

(1) $$\begin{array}{c} u_{0},e_{1},u_{1},\ldots ,u_{l-1},e_{l},u_{l}. \end{array}$$

With terms interchangely contained in vertex set *V*(*H*) and edge set *E*(*H*), where *u*_*j*−1_ and *u*_*j*_ are connected by an edge *e*_*j*_, 1 ≤ *j* ≤ *l*. A walk is *closed* if its originating and ending vertices coincide and is a *trail* if its all edges are pairwise different. A trail passing all edges of *H* is an *Eulerian trail*, and an *Eulerian tour* is a closed Eulerian trail.

We refer to [1] for terms and notation not mentioned. The degree of *u* in a graph *H* is written as *d*(*u*), and the minimum degree of *H* is denoted by *δ*(*H*). We use *A*(*v*) to denote the set of all arcs outgoing from *v*, and *A*(*G*) to represent the set of all arcs of *G*.

In the following we give the formal definition of the 3-arc graph of a graph *H*.

**Definition 1 .**

Let *H* be a graph. The 3-arc graph *X*(*H*) of *H*, is the graph with vertex set *A*(*H*) and all edges connecting *xy* and *vw* whenever (*y*, *x*, *v*, *w*) is a 3-arc of *H*.

**Figure 1:**
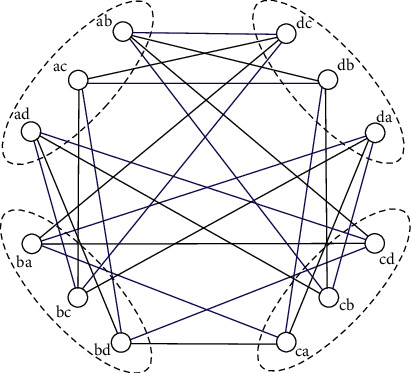
The 3-arc graph of *K*_4_.

We illustrate the definition of this construction by depicting the 3-arc graph of complete graph *K*_4_ on four vertices *a*, *b*, *c* and *d* (see Figure 1). For instance, both (*a*, *b*, *c*, *d*) and (*a*, *b*, *c*, *a*) are 3-arcs in *K*_4_. The first one defines the adjacent relation between arcs *ba* and *c*  *d*, and the latter gives rise to the adjacency between *ba* and *ca*.

It is not hard to see that *X*(*H*) is not directed with 2|*E*(*H*)| vertices and ∑_{*x*, *y*}∈*E*(*H*)_(*d*(*x*) − 1)(*d*(*y*) − 1) edges. It is shown that *X*(*H*) can actually be obtained from *L*(*H*) of *H* by the next procedures: divide every vertex {*x*, *y*} of *L*(*H*) into two, i.e., *xy* and *yx*; for every pair of vertices {*a*, *b*}, {*x*, *y*} of *L*(*H*) that are at a distance 2 away in *L*(*H*), say, *a* and *x* are adjoined in *H*, connect *ab* and *xy*. For more recent work in this direction, see [6]. Specifically, in [7], it is established that all connected graphs contain a Hamilton cycle.

In 1975, Sheehan proposed the following famous conjecture:

**Conjecture 1 (Sheehan [8]).**

Each Hamiltonian graph that is regular of degree 4 consists of no less than two Hamilton cycles.

Motivated by Sheehan's Conjecture and the work [7], we study the Hamiltonian decomposition problem for 3-arc graphs. We show every connected 3-arc graph contains a second Hamilton cycle, and the 3-arc graphs of a family of cubic graphs have Hamiltonian decompositions. As a corollary, we demonstrate that the 3-arc graph of a bipartite cubic graph includes one Hamiltonian decomposition.

To confirm Sheehan's conjecture for 3-arc graphs, we actually prove a stronger result which asserts that any connected 3-arc graph contains no less than two Hamilton cycles. As the 3-arc graph of a cubic graph is 4-regular. We show further that each 3-arc graph of a cubic graph in a certain family has a Hamiltonian decomposition.

The primary results are presented as follows.

**Theorem 1 .**

Let *G* be with a maximum degree Δ and have a connected 3-arc graph *X*(*G*). Then the number of Hamilton cycles of *X*(*G*) is no less than the following:

(2) $$\begin{array}{c} \left\{\begin{array}{cc} 2, & if\;\Delta =3\;or\;4;\\ {\left(\frac{{\left(\Delta -3\right)}^{\Delta -3}}{{\left(\Delta -2\right)}^{\Delta -4}}\right)}^{\Delta }, & \;if\;\geq 5. \end{array}\right.. \end{array}$$

**Remark 1 .**

Since ((Δ − 3)^Δ−3^/(Δ − 2)^Δ−4^)^Δ^=((Δ − 2)1/(1+1/Δ−3)^Δ−3^)^Δ^, (1+1/Δ−3)^Δ−3^ is increasing and

(3) $$\begin{array}{c} {\text{lim}}_{\Delta ⟶\infty }{\left(1+\frac{1}{\Delta -3}\right)}^{\Delta -3}=e. \end{array}$$

**Theorem 2 .**

Suppose *G* is a connected cubic graph. If *G* contains an even IS-C decomposition, then *X*(*G*) has a Hamiltonian decomposition.

## 2. Proof of Theorems 1 and 2

We need the following notation and definitions.

A *cactus* is a graph in which any two cycles have at most one vertex in common. In particular, a tree is a special cactus. A cycle is said to be an *odd* (resp., *even*) *cycle* if it consists of an odd (resp., even) number of edges.

**Definition 2 .**

Let (*A*, *B*) be a partition (i.e., *V*(*G*) : =*A* ∪ *B* and *A*∩*B*=∅) of *V*(*G*). (*A*, *B*) is said to be an independent set-cactus (IS-C) decomposition of *G* if in *G* the set *A* induces an empty graph and *B* induces a (connected) cactus.

Note that not every graph has an IS-C decomposition. For example, the complete graph on five or more vertices has no IS-C decomposition.

Since trees are acyclic, as a convention, we define the length of each cycle (which does not exist) of a tree to be 0. A cactus is called *even* if the length of each cycle is even. We treat a tree as an even cactus. An IS-C decomposition is called *even* if every cycle in the cactus is even.

**Lemma 1 .**

Each connected cubic graph has an IS-C decomposition.

Proof. Suppose *G* is a connected cubic graph. If every two cycles of *G* share one or less common vertex, then *G* itself is a cactus. Clearly, an IS-C decomposition, (∅, *V*(*G*)), say, exists.

Suppose that *G* has two distinct cycles sharing two or more common vertices. We construct an IS-C decomposition for *G* as follows: choose any two cycles *C*_1_, *C*_2_ that share at least two common vertices. Denote the maximal common path occurring on both *C*_1_ and *C*_2_ by *P* : *x*,…, *x*′*P* : *x*,…, *x*′; that is, any vertex not on *P* occurs on at most one of the two cycles *C*_1_ and *C*_2_. Then delete *x* from *G* and put it into the set *A* (*A* takes the role of the independent set). Note that each neighbour of *x* now has degree strictly less than 3, and hence occurs on at most one cycle in *G* − *x*. So, the resultant graph *G* − *x* is connected. To simplify notation, denote again by *G* the graph resulting after deleting *x*. If *G* still has two cycles *C*_3_, *C*_4_ that share at least two common vertices. Denote the maximal common path occurring on both *C*_3_ and *C*_4_ by *y*,…, *y*′. Note that *y*, *y*′ ∉ *N*(*x*). Delete *y* and put it into *A*. Continue this process until the resultant graph becomes a cactus.

Since, at each stage, the set *A* remains to be independent and the resulting graph stays to be connected, eventually (*A*, *V*(*G*) − *A*) becomes a desired IS-C decomposition.

Though every cubic graph has an IS-C decomposition, not every cubic graph has an even IS-C decomposition. For instance, *K*_4_ has no even IS-C decomposition.

A 2-trail (*u*, *x*, *v*) with the middle term *x* is said to be a *visit tox*. If *u* ≠ *v*, then (*u*, *x*, *v*) and (*v*, *x*, *u*) are viewed as different visits to *x*. When we (*u*, *x*, *v*)(*v*, *x*, *u*)are not concerned about the directions of and , or its orientation is unknown, we denote [*u*, *x*, *v*]. The following operation regarding two parallel edges will be needed.

**Definition 3 .**

Let *e*_1_, *e*_2_ be parallel edges joining two adjacent *u*, *v* of *G*^*∗*^, let *e*_*i*_ be covered by a closed trail *C*_*i*_ of length at least 4, *i*=1,2. (It may happen that *C*_1_=*C*_2_). Then one of *C*_1_(*u*) ∪ *C*_2_(*u*) and *C*_1_(*v*) ∪ *C*_2_(*v*), say *C*_1_(*v*) ∪ *C*_2_(*v*), contains two visits with one covering *e*_1_ and the other covering *e*_2_. Denote these two visits by *p*_1_=[*u*, *e*_1_, *v*, *e*_3_, *v*_1_] and *p*_2_=[*u*, *e*_2_, *v*, *e*_4_, *v*_2_], where *v*_1_, *v*_2_ are two neighbors of *v* other than *u*, and, *e*_3_, *e*_4_ are edges between *v* and *v*_1_, *v*_2_ respectively. Split the two 2-trails *p*_1_, *p*_2_ at *v* and reconnect *e*_1_, *e*_2_ with *e*_4_, *e*_3_, respectively. We call this the edge-shift operation of *C*_1_, *C*_2_ with respect to *e*_1_, *e*_2_, and denote the resultant trail(s) by *C*(*C*_1_, *C*_2_; *e*_1_, *e*_2_), or simply *C*(*C*_1_; *e*_1_, *e*_2_) if *C*_1_=*C*_2_.

**Remark 2 .**

A few comments on Definition 3 are ready:

1. If *C*_1_=*C*_2_, *C*(*C*_1_; *e*_1_, *e*_2_) remains to be a single closed trail if and only if the orientations of *e*_1_ and *e*_2_ are reverse to each other in *C*_1_; in this case, this operation is, in fact, the edge version of the bow-tie operation (see, Definition 5 in [7]).
2. If *C*_1_=*C*_2_, *C*(*C*_1_; *e*_1_, *e*_2_) is a set of two closed trails if and only if the orientations *e*_1_ and *e*_2_ are the same between *u* and *v* in *C*_1_.
3. All edges covered by *C*_1_, *C*_2_ are covered by *C*(*C*_1_, *C*_2_; *e*_1_, *e*_2_).
4. After the edge-shift operation above, the three terms involved in *p*_1_, *p*_2_ are swapped. That is, *p*_1_ is transformed into [*u*, *v*, *v*_2_], and *p*_2_ is transformed into [*u*, *v*, *v*_1_]. All other visits induced are kept or with orientation inverted.

**Proof**

Suppose *G* is a graph with *X*(*G*) connected. Clearly, Δ(*G*) ≥ 3. Denote the set of degree-2 vertices of *G* by *S*_2_, the multigraph gained from *G* by doubling its every edge by *G*^*∗*^.

Suppose that Δ=3. Since *X*(*G*) is connected, by [7], *X*(*G*) is Hamiltonian, and every vertex in *G* has degree 2 or 3, *S*_2_ is independent, and *G* − *S*_2_ is connected. Further, it can be observed that *G* − *S*_2_ contains at least 2 vertices. Suppose *u*, *v* are two adjacent vertices in *G* − *S*_2_, *u*′ is a neighbor of *u* different from *v*, and *x*, *y* are two distinct neighbors of *v* not equal *u* (it may occur that one of *x*, *y* equals *u*′). Denote *P*_1_ : *u*′, *u*, *v*, *x* and *P*_2_ : *u*′, *u*, *v*, *y*.

Let *C*_1_, *C*_2_ be two Eulerian tours of *G*^*∗*^ obtained without violating the condition (1) in Section 3 of [7], from extending *P*_1_, *P*_2_ to cover each edge of *G*^*∗*^, respectively. Apply the bow-tie operation (Definition 5 in [7]) when necessary to process *C*_*j*_ in such a way that the bipartite graph *H*_*C*_*j*_′_(*z*) (Definition 3 in [7]) with respect to the resultant Eulerian tour *C*_*j*_′ has a perfect matching and the path *P*_*j*_ is a segment of *C*_*j*_′, *j*=1,2. Note that both *C*_1_′ and *C*_2_′ exist. Consider *C*_1_. If *H*_*C*_1__(*v*) has no perfect matching, then *C*_1_(*v*) contains twin visits by Lemma 1 in [7], that is, *C*_1_(*v*)={[*u*, *v*, *x*], [*u*, *v*, *x*], (*y*, *v*, *y*)}. Apply the bow-tie operation to *C*_1_ with regard to (*y*, *v*, *y*) and anyone of the twin visits [*u*, *v*, *x*] that is not occurring on *P*_1_, we get a new Eulerian tour *C*_1_′=*C*_1_([*u*, *v*, *x*], (*y*, *v*, *y*)), and one can observe that *H*_*C*_1_′_(*v*) is a perfect matching of 3 independent edges. The bow-tie operation can be performed similarly when necessary at *u* to produce a new Eulerian tour *C*_1_′ such that *H*_*C*_1_′_(*u*) has a perfect matching and the path *P*_*j*_ is maintained unchanged. It is analogous to showing that *C*_2_′ exists.

Let *T*_1_, *T*_2_ be two Hamilton cycles of *X*(*G*) derived from *C*_1_′, *C*_2_′, respectively. Denote the 3rd neighbor of *u* other than *u*′, *v* by *w*. Then one can observe that *uw* is connected to *u*′*z*, *vy* in *T*_1_, while connected to *u*′*z*′, *vx* in *T*_2_, where *z*, *z*′ ∈ *N*(*u*′) − {*u*} are not necessarily distinct. Since {*u*′*z*, *vy*} ≠ {*u*′*z*′, *vx*}, *T*_1_ and *T*_2_ are different Hamilton cycles of *X*(*G*).

Suppose that Δ ≥ 4. Suppose *x* is the maximum-degree vertex, *C* an Eulerian tour of *G*^*∗*^ with the property that *H*_*C*_(*z*) contains a perfect matching for each *z* (such a *C* can be achieved by applying the bow-tie operations successively when needed). Denote $\hat{C}\left(x\right)$ the family of heavy visits to *x* (that is, each element of $\hat{C}\left(x\right)$ is of the form [*w*, *x*, *w*], where *w* is a neighbor of *x*). By performing a series of bow-tie operations, we will transform each visit of $\hat{C}\left(x\right)$ into a new visit containing three pairwise distinct terms.

To simplify notation we will still use *C* to denote the new Eulerian tour produced by applying an operation. If $\left|\hat{C}\left(x\right)\right|\geq 2$, choose a pair of visits *p*_1_, *p*_2_ of $\hat{C}\left(x\right)$, and apply bow-tie operation to *C* with regarding to *p*_1_, *p*_2_. Then in the new Eulerian tour *C*(*p*_1_, *p*_2_), the pair *p*_1_, *p*_2_ is transformed into two visits with each containing three pairwise distinct terms. Thus, each time of applying the bow-tie operation to *C* with regard to two elements of $\hat{C}\left(x\right)$ deducts the number of $\left|\hat{C}\left(x\right)\right|$ by two. Apply this operation until $\left|\hat{C}\left(x\right)\right|\leq 1$. If $\left|\hat{C}\left(x\right)\right|=0$, we are done. Suppose $\hat{C}\left(x\right)=\left\{\left[w,x,w\right]\right\}$. Let *p*=[*y*, *x*, *z*] be an arbitrary visit outside $\hat{C}\left(x\right)$. Apply the same operation to *C* with regard to [*w*, *x*, *w*] and *p*. Again, [*w*, *x*, *w*] can be eliminated without producing a new heavy visit. Eventually, we get an Eulerian tour, denoted again by *C*, of *G*^*∗*^ such that each element of *C*(*x*) contains three pairwise distinct terms. Then in *H*_*C*_(*x*), each visit of *C*(*x*) is adjacent to Δ − 2 arcs of *A*(*x*), and each arc of *A*(*x*) is adjacent to Δ − 2 visits of *C*(*x*). That is, *H*_*C*_(*x*) is a (Δ − 2)-regular bipartite graph.

If Δ=4, *H*_*C*_(*x*) is 2-regular, hence contains two distinct perfect matchings. Suppose that Δ ≥ 5, and denote by *M*_1_, *M*_2_,…, *M*_*i*_ the set of all perfect matchings of *H*_*C*_(*x*). Schrijver [9] states that every bipartite graph regular of degree *k* with 2*n* vertices contains at least ((*k* − 1)^*k*−1^/*k*^*k*−2^)^*n*^ perfect matchings. Thus, we have the following:

(4) $$\begin{array}{c} i\geq {\left(\frac{{\left(\Delta -3\right)}^{\Delta -3}}{{\left(\Delta -2\right)}^{\Delta -4}}\right)}^{\Delta }. \end{array}$$

For every vertex *u* ≠ *x* in *G*, fix a perfect matching of *H*_*C*_(*u*). As in the Proof of Theorem 1 in [7], a Hamilton cycle of *X*(*G*) can be derived from *C* by using the fixed perfect matchings of all bipartite graphs *H*_*C*_(*v*) with *v* ≠ *x*, together with every single perfect matching *M*_*j*_ of *H*_*C*_(*x*), 1 ≤ *j* ≤ *i*. Note that corresponding to distinct *M*_*j*_, *M*_*j*′_ of *H*_*C*_(*x*), the derived Hamilton cycles of *X*(*G*) are also distinct. Therefore, *X*(*G*) has *i* different Hamilton cycles and the result follows.□

Suppose *G* is a graph containing no isolates. If *X*(*G*) is connected and 4-regular, then clearly *G* has a maximum degree of no less than 3. Hence by Theorem 1, we have the next result, which verifies Sheehan's conjecture for 3-arc graphs.

**Corollary 1 .**

Every 4-regular 3-arc graph has no less than two Hamilton cycles.

**Proof**

Suppose *G* is a connected cubic graph and (*A*, *B*) an even IS-C decomposition of *G*. To simply notation, we employ *B* to represent the cactus induced by *B*.

Suppose *G*^*∗*^ is the multigraph achieved from *G* by doubling its all edges, *B*^*∗*^ the multigraph achieved from *G*^*∗*^ by deleting all edges joining some vertex of *A*. Equivalently, *B*^*∗*^ is the multigraph obtained from *B* by doubling each of its edges.

Then *B*^*∗*^ is Eulerian. Assume *C*′ is an Eulerian tour of *B*^*∗*^ , so that *C*′(*x*) contains no heavy visit for each *x* ∈ *B* with 2 or 3 neighbors in *B*. Note that such a *C*′ can be obtained from any Eulerian tour of *B*^*∗*^ by applying a succession of bow-tie operations when necessary.

We next extend *C*′ to an Eulerian tour of *G*^*∗*^ as follows: for each vertex *x* ∈ *B* with degree 1 in *B*, let *x*′ be the only neighbor of *x* in *B*, and *x*_1_, *x*_2_ the other two distinct neighbors of *x* in *A*. Then *C*′(*x*)={(*x*′, *x*, *x*′)}. We extend *C*′ at *x* to cover the four edges between *x*_1_, *x*_2_ and *x* such that the new visit decomposition at *x* is {[*x*′, *x*, *x*_1_], [*x*_1_, *x*, *x*_2_], [*x*_2_, *x*, *x*]′}. In other words, we insert the trail *x*, *x*_1_, *x*, *x*_2_, *x* into *C*′ via the midvertex of the visit (*x*′, *x*, *x*′). For each vertex *x* ∈ *B* with degree 2 in *B*, let *x*_1_ be the unique neighbor of *x* in *A*, and *x*′, *x*′′ the other two distinct neighbors of *x* in *B*. Then *C*′(*x*)={[*x*′, *x*, *x*′′], [*x*′, *x*, *x*′′]} by the assumption on *C*′. We extend *C*′ at *x* to cover the two edges between *x*_1_ and *x* such that the new visit decomposition is {(*x*′, *x*, *x*_1_), (*x*_1_, *x*, *x*′′), [*x*′, *x*, *x*′′]}. Applying this extension to every vertex of *B* that has degree 1 or 2, we obtain an Eulerian tour of *G*^*∗*^, denoted *C*.

By the way *C* is extended, one may observe that *C*(*x*) consists of no heavy visit if *x* ∈ *B*, and *C*(*x*) contains only heavy visits if *x* ∈ *A*. In particular, the bipartite graph *H*_*C*_(*x*) is a set of three independent edges if *x* ∈ *B*, and *H*_*C*_(*x*)≅*C*_6_ if *x* ∈ *A*. Thus, in both cases *H*_*C*_(*x*) has a perfect matching, and, a Hamilton cycle of *X*(*x*), denoted by *C*^*X*^, can be derived from *C*.

Since *G* is cubic, for each pair of adjacent *a*, *b* of *G*, by Definition 1, the subgraph *X*(*a*, *b*) induced by vertices in *A*(*a*) ∪ *A*(*b*) in *X*(*G*) is isomorphic to *K*_2,2_.

For any two vertices *a*, *b* adjacent in *B*, let *a*_1_, *a*_2_ be two distinct neighbours of *a* other than *b*, and *b*_1_, *b*_2_ be two distinct neighbours of *b* other than *a*. Since each of *C*(*a*) and *C*(*b*) contains no heavy visit, we have *C*(*a*)={[*a*_1_, *a*, *b*], [*a*_2_, *a*, *b*], [*a*_1_, *a*, *a*_2_]} and *C*(*b*)={[*b*_1_, *b*, *a*], [*b*_2_, *b*, *a*], [*b*_1_, *b*, *b*_2_]}. Let *e*_1_, *e*_2_ be parallel between *a* and *b* in *B*^*∗*^. Then each of *e*_1_, *e*_2_ is contained in a visit of *C*(*a*) and a visit of *C*(*b*). Assume w.l.o.g. that [*a*_1_, *a*, *b*], [*b*_1_, *b*, *a*] contain *e*_1_ and [*a*_2_, *a*, *b*], [*b*_2_, *b*, *a*] contain *e*_2_. Then each of *a*_1_, *a*, *b*, *b*_1_ and *a*_2_, *a*, *b*, *b*_2_ is a segment of length 3 of *C*. We may define each of these two segments as a *visit* to the edge {*a*, *b*} of *B*, and denote them as [*a*_1_, *a*, *b*, *b*_1_], [*a*_2_, *a*, *b*, *b*_2_], respectively. From the definition of *B*^*∗*^, each edge, and hence {*a*, *b*}, of *B* is visited twice by *C* of *B*^*∗*^. Represent by *C*({*a*, *b*}) the set of two visits of *C* to the edge {*a*, *b*}, i.e., *C*({*a*, *b*})=[*a*_1_, *a*, *b*, *b*_1_], [*a*_2_, *a*, *b*, *b*_2_].

Then, each element of *C*({*a*, *b*}) is a trail of length 3 which corresponds exactly to one edge of *X*(*a*, *b*) covered by *C*^*X*^. And, the two visits of *C*({*a*, *b*}) are in fact corresponding to two independent edges of *X*(*a*, *b*), namely, {*aa*_2_, *bb*_2_}, {*aa*_1_, *bb*_1_}, which are covered by *C*^*X*^. This means that *C*^*X*^ covers exactly one perfect matching (two independent edges) of *X*(*a*, *b*) and leaves the other perfect matching uncovered by noting that *X*(*a*, *b*)≅*K*_2,2_ contains two perfect matchings.

Apply the edge-shift operation (Definition 3) to *C* with respect to the 2 parallel edges connecting *a* and *b*, denote the resulted trail(s) by *C*_0_. Then *C*({*a*, *b*}) is transformed into *C*_0_{*a*, *b*}={[*a*_1_, *a*, *b*, *b*_2_], [*a*_2_, *a*, *b*, *b*_1_]}. If *C*_0_ stays as an Eulerian tour of *G*^*∗*^, one can observe that any Hamilton cycle *C*_0_^*X*^, derived from *C*_0_, will cover the pair of independent edges of *X*(*a*, *b*) which are not covered by *C*^*X*^.

The following claim shows that we can apply the edge-shift operations to every pair of parallel edges of *B*^*∗*^ once and still get an Eulerian tour of *G*^*∗*^.

Claim. There are a series of edge-shift operations such that to every pair of parallel edges of *B*^*∗*^ , exactly one operation is performed and the resultant trail is an Eulerian tour of *G*^*∗*^.

Proof of the Claim. First consider an edge {*x*, *y*} belonging to none of the cycles of *B*, then {*x*, *y*} is a bridge of *B*. Let *e*_1_, *e*_2_ be the two parallel edges between *x* and *y* in *B*^*∗*^. Then the orientations of *e*_1_, *e*_2_ are opposite to each other in *C*, by (1) of Remark 2, *C*(*C*; *e*_1_, *e*_2_) stays as an Eulerian tour of *B*^*∗*^. Thus, we can apply the edge-shift operation to each pair of parallel edges of *B*^*∗*^ joining two end-vertices of a bridge of *B*. To simplify notation, denote again by *C* the Eulerian tour of *B*^*∗*^ after processing all such pairs of parallel edges.

Next we process edges on cycles in the cactus *B*. Since Δ(*B*) ≤ 3, any two distinct cycles in *B* are vertex- and edge-disjoint. We process these cycles one by one.

Let *L*=*x*_1_, *x*_2_,…, *x*_*l*_, *x*_1_ be an arbitrary cycle of *B*, where *l* ≥ 4 is even. Since every vertex has degree 3 in *G*, each *x*_*j*_ has a neighbor, denoted by *x*_*j*_′, where 1 ≤ *j* ≤ *l*. Note that each edge {*x*_*j*_, *x*_*j*_′} is a bridge of *B*. Denote by *L*_*j*_ the component containing *x*_*j*_′ after deleting the edge {*x*_*j*_, *x*_*j*_′}. If *C* proceeds along some visit (*x*_*j*±1_, *x*_*j*_, *x*_*j*_′) into *L*_*j*_, *C* must return back to the component containing the cycle *L* via the visit (*x*_*j*_′, *x*_*j*_, *x*_*j*∓1_) immediately after *C* finishing its all traverses in *L*_*j*_. We can shrink the traverse of *C* in *L*_*j*_ as a simple visit to *x*_*j*_ as follows: suppose *C* proceeds its traverses in *L*_*j*_ using the following trail:

(5) $$\begin{array}{c} T^{\prime }:x_{j-1},x_{j},x_{j}^{\prime },\ldots ,x_{j}^{\prime },x_{j},x_{j+1}. \end{array}$$

We can first shrink this trail into the visit (*x*_*j*−1_, *x*_*j*_, *x*_*j*+1_) to *x*_*j*_, and this visit could be restored back to its original form *T*′ above after the processing of the edges of *L*. After shrinking all such traverses of *C* in *L*_*i*_, where 1 ≤ *j* ≤ *l*, we get an Eulerian tour $\tilde{C}$ of the multigraph *L*^*∗*^ (*L*^*∗*^ is the multigraph obtained from the cycle by doubling each of its edges). Equivalently, $\tilde{C}$ is a single walk that covers each edge of the simple cycle *L* twice. Then, for each edge *e*_*j*_ : ={*x*_*j*_, *x*_*j*+1_} of *L*, denoted by *e*_*j*_, *e*_*j*_′, the two parallel edges between *x*_*j*_ and *x*_*j*+1_.

Apply the edge-shift operation to $\tilde{C}$ with respect to *e*_*j*_, *e*_*j*_′. Since the orientations of *e*_*j*_, *e*_*j*_′ in $\tilde{C}$ are the same, $C\left(\tilde{C};e_{j},e_{j}^{\prime }\right)$ is a set of two closed trails, *C*_1_, *C*_2_, say. One can observe that the trail covering *e*_*j*+1_ and that covering *e*_*j*+1_′ are distinct, where *e*_*j*+1_, *e*_*j*+1_′ are two parallel edges between *x*_*j*+1_ and *x*_*j*+2_. After this operation, we immediately apply the edge-shift operation to *C*_1_, *C*_2_ with respect to *e*_*j*+1_, *e*_*j*+1_′. Then, this operation will concatenate *C*_1_ and *C*_2_ together, that is, *C* (*C*_1_, *C*_2_; *e*_*j*+1_, *e*_*j*+1_′) will become again an Eulerian tour of *L*^*∗*^.

Continue applying this operation to the pairs (*e*_*j*+2_, *e*_*j*+2_′), (*e*_*j*+3_, *e*_*j*+3_′),…, (*e*_*j*−1_, *e*_*j*−1_′)(*e*_*j*+2_, *e*_*j*+2_′), (*e*_*j*+3_, *e*_*j*+3_′),…, (*e*_*j*−1_, *e*_*j*−1_′); we finally obtain an Eulerian tour of *L*^*∗*^, denoted again by $\hat{C}$. Then we restore $\hat{C}$ back to an Eulerian tour of *B*^*∗*^.

Using this procedure to process all cycles of *B*, we finally obtain an Eulerian tour of *G*^*∗*^ after performing exactly one operation to every pair of parallel edges of *B*^*∗*^. The claim follows.□

Denote by $\bar{C}$, the Eulerian tour of *G*^*∗*^ produced after applying the edge-shift operation exactly once to each pair of parallel edges connecting two adjacent vertices of *B*.

Consider every edge *e* with one end-vertex *x* ∈ *A* (then *e* is not in *B*). Then all neighbours of *x* lie in *B*. Denote *N*(*x*)={*x*_1_, *x*_2_, *x*_3_}, *e*={*x*, *x*_1_} and *N*(*x*_1_)={*x*, *z*, *z*′}, where *x*_1_, *x*_2_, *x*_3_ ∈ *B*. Then, by the construction of *C*,

(6) $$\begin{array}{c} C\left(x\right)=\left\{\left(x_{1},x,x_{1}\right),\left(x_{2},x,x_{2}\right),\left(x_{3},x,x_{3}\right)\right\}. \end{array}$$

In *X*(*x*_1_, *x*), such a heavy visit (*x*_1_, *x*, *x*_1_) is exactly corresponding to two adjacent edges of *X*(*x*_1_, *x*). For example, in this instance, if *C* enters *x* via *z*, *x*_1_ and leaves via *x*_1_, *z*′ to complete the visit (*x*_1_, *x*, *x*_1_)(*x*_1_, *x*, *x*_1_); then, it 1-1 corresponds to the two edges {*x*_1_*z*′, *xt*}, {*xt*, *x*_1_*z*} , which is a 2-path in *X*(*x*_1_, *x*) (also in the Hamiltonian cycle *C*^*X*^), where *t* is either *x*_2_ or *x*_3_. Note that the other two edges in *X*(*x*_1_, *x*) uncovered by *C*^*X*^ will be available in the second Hamilton cycle ${\bar{C}}^{X}$.

We do not apply any operation on edges *e*={*x*, *x*_1_} with one end-vertex in *A*. We can just use the two uncovered edges in the new Hamilton cycle when constructing it. Equivalently, for each *x* ∈ *A*, the perfect matching $H_{\bar{C}}\left(x\right)$ employed in deriving ${\bar{C}}^{X}$ is the one that shares no common edge with *H*_*C*_(*x*).

Then, ${\bar{C}}^{X}$ is a Hamilton cycle sharing no edges with *C*^*X*^, the proof is completed.□

**Corollary 2 .**

Suppose *G* is cubic. If *G* has a stable set such that whose removal leaves *G* a tree, then *X*(*G*) has a Hamiltonian decomposition.

Since every cubic graph has an IS-C decomposition, every bipartite cubic graph has an even IS-C decomposition. We have the following.

**Corollary 3 .**

The 3-arc graph of any bipartite cubic graph has a Hamiltonian decomposition.

Let *P* be the Petersen graph. It is not difficult to find a stable set of *P* consisting of three vertices whose removal leaves *P* a tree. Let *G* be an *n*-prism, *n* ≥ 3. Then, *G* is isomorphic to the Cartesian product *C*_*n*_□*K*_2_. Denote *V*(*G*)=*V*(*C*) ∪ *V*(*C*′), where *V*(*C*)={*v*_1_, *v*_2_,…, *v*_*n*_}, *V*(*C*′)={*v*_1_′, *v*_2_′,…, *v*_*n*_′}, and, each of *V*(*C*) and *V*(*C*′) induces an *n*-cycle. The edge set of *G* is *E*(*C*) ∪ *E*(*C*′) ∪ {{*v*_*i*_, *v*_*i*_′} : *i*=1,2,…, *n*}. Set *S* : ={*v*_2*j*_ : *j*=1,2,…, ⌊*n*/2⌋}. If *n* is even, then (*S*, *V*(*G*) − *S*) is an even IS-C decomposition of *G* with *V*(*G*) − *S* inducing a unicyclic graph; if *n* is odd, then (*S* ∪ {*v*_1_′}, *V*(*G*) − (*S* ∪ {*v*_1_′})) is an even IS-C decomposition of *G* with *V*(*G*) − (*S* ∪ {*v*_1_′}) inducing a tree. By applying 2 and 3, we obtain the following example:

**Example 1 .**

(1) The 3-arc graph of Petersen graph has a Hamiltonian decomposition; (2) The 3-arc graph of the *n*-prism has a Hamiltonian decomposition.

**Remark 3 .**

The condition in Theorem 2 is sufficient but not necessary. For example, *K*_4_ does not have an even IS-C decomposition but *X*(*K*_4_) is still Hamiltonian decomposable. In *X*(*K*_4_), see Figure 1, each of the two sets of bold edges (in color black) and thin edges (in color blue) forms a Hamilton cycle [10].

## Data Availability

The findings of this study are supported by the rigorous proofs which are included within the paper.
